# Evaluation of Anti-Inflammatory Activity and *In-vitro *Antioxidant Activity of *Indian Mistletoe*, the Hemiparasite *Dendrophthoe falcate *L. F. (Loranthaceae)

**Published:** 2011

**Authors:** Satish Patil, Sneha Anarthe, Ram Jadhav, Sanjay Surana

**Affiliations:** a *Department of Bioecology and Phytochemistry, R.C. Patel College of Pharmacy, Shirpur, Maharashtra, India.*; b *Department of Pharmacognosy, Gokaraju Rangaraju College of Pharmacy, Hyderabad , And hra Pradesh, India.*

**Keywords:** *Dendrophthoe falcate*, In-vitro antioxidant activity, Anti- inflammatry activity, Methanolic extracts

## Abstract

Methanolic and aqueous extracts of *Dendrophthoe falcata* Linn. leaves which belongs to the Loranthaceae family, were evaluated through DPPH (1, 1-diphenyl -2-picryl-hydrazyl), antilipid peroxidation and nitric oxide scavenging methods to assess the antioxidant activity. Methanolic and aqueous extracts of *Dendrophthoe falcata* leaves were also evaluated for their anti-inflammatory activity by carrageenan and cotton pellet induced granuloma tests for their effect on the acute and chronic phase inflammation models in rats. It was found that the methanolic extract of *Dendrophthoe falcata* leaves demonstrates potent antioxidant activity as compared to aqueous extraction of *Dendrophthoe falcata *leaves for DPPH (1, 1-diphenyl-2-picryl-hydrazyl) radical scavenging, anti-lipid peroxidation and nitric oxide scavenging activity respectively (having IC_50_ value 77.8, 79.36 and 86.2, 144, 87, 104). The maximum inhibition for aqueous extract of *Dendrophthoe falcata *leaves (30.95%) and methanolic extract of *Dendrophthoe falcata* leaves (23.41%) were obtained at a dose of 300 mg/Kg after 4h of drug treatment in carrageenan induced paw edema, whereas diclofenac sodium (standard drug) produced 42.85% inhibition. In the chronic model (cotton pellet induced granuloma), aqueous extracts of *Dendrophthoe falcata* leaves and methanolic extracts of *Dendrophthoe falcata *leaves (at doses of 300 mg/Kg), phenylbutazone as standard drug showed decreased formation of granuloma tissue by 51%, 48%, 53% respectively. In addition, the total phenolic and flavonoid content of aqueous extracts of *Dendrophthoe falcata *leaves and methanolic extracts of *Dendrophthoe falcata* leaves were found to be 2.12 % w/w, 4.39 % w/w, 0.31 mg/g and 0.85 mg/g respectively. Thus the results indicate that methanolic and aqueous extracts of *Dendrophthoe falcata*leaves on animal models have potent anti-inflammatory and in-vitro antioxidant effects.

## Introduction

The genus Dendrophthoe is evergreen, shrubby, partial parasites, distributed in the tropical and sub-tropical regions of the old world. The whole parasitic plant *Dendrophthoe falcata* is used in indigenous system of medicine ascooling, bitter, astringent, aphrodisiac, narcotic and diuretic and is also useful in pulmonary tuberculosis, asthma, menstrual disorders, swelling wounds, ulcers, renal and vesicle calculi and vitiated conditions of kapha and pitta. The decoction of *Dendrophthoe falcata* is used by women as antifertility agent. The *Dendrophthoe falcata* also have anticancer activity (1). *Dendrophthoe falcate* that is branched angiospermic hemiparasite, was most frequently observed on hosts* Mangifera indica*(Anacardiaceae), *Melia azadirecta* (Meliaceae) and *Psidium guayava *(Myrtaceae). Barks of *Dendrophthoe falcata* are grey, its leaves are thick, coriaceous, much variable in shape usually opposite 7.5 to 18 by 2-10 cm, and its flowers are stout, unilateral racemes often two from an axil pedicle. The flowers are ovate sub-acute, concave and scarlet or orange or less commonly pink or white in colour. Anthers are linear, equal in length to the free portion of the filament. Berries of *Dendrophthoe falcata* are 8-13 mm long ovoid oblong, pink, smooth crowned by a cup- shaped calyx (2). The genus *Dendrophthoe *comprises of 20 species and about 7 species are found in India. Members of genus *Dendrophthoe*are reported to have anti-oxidant, anti-microbial, anticancer,antidiabetic (3), anti-lithiatic and antihypertensive activity (4). 

Angiospermic parasitic plant *Dendrophthoe falcata*, reported to contain biologically active substances such as flavonoid quercetin (5), tannins, *β*-sitosterol, *β*-amyrin, oleanolic acid (6, 7). 

The present study was undertaken to investigate the *in-vitro* antioxidant and anti-inflammatory activity of *Dendrophthoe falcata *leaves.

## Experimental

*Materials*

The leaves of *Dendrophthoe falcata*parasitic on *Mangifera indica* (Anacardiaceae) were collected from western ghat region of maharashtra (India) in February 2005. The *Dendrophthoe falcata* plant specimen was authenticated from botanical survey of india, pune (Voucher specimen no. PSH-1). The air-dried leaves of *D. falcata* were pulverized and the powdered material was extracted with methanol (80 %) and chloroform water by cold maceration at room temperature for seven days, chloroform water is used to avoid the fungal growth during the extraction (3.5 mL chloroform in 1000 mL of distilled water). The methanolic and aqueous extracts of *Dendrophthoe falcata* leaves were concentrated on a rotary vacuum evaporator at reduced pressure, which gave a yield (4.16 and 9.42 % w/w). The proximate phytochemical analysis of methanol and aqueous extracts of *Dendrophthoe falcata *leaves shows presence of flavonoids, proteins and carbohydrates.

*Animals*

Wistar rats, of either sex, weighing 180–250 g were used. They were housed under standard conditions of temperature (23 ± 2°C), humidity and dark-light cycle. They were given standard diet and water ad libitum. All the animals were carefully monitored and maintained in accordance with CPCSEA guidelines on control and supervision of experimental animals for 15 days. The ethical clearance was obtained from the Institutional Animal Ethics Committee (Approval no.651/02/c/CPCSEA) before the experiment.

*Anti-inflammatory activity*

*Carrageenan-induced rat paw edema*

The anti-inflammatory activity was evaluated using Carrageenan-induced rat paw edema according to method described by Winter *et al.*(8). The animals were starved overnight before the experiment to ensure uniform hydration. Fasting rats were divided into eight groups each carrying six animals. Group I served as a control and received Tween 80 (5 mL/Kg) of 2% w/v, orally. Diclofenac sodium (5 mg/Kg, p.o.) was administered to group II as standard. Groups III–VIII received the aqueous and methanolic extracts of *Dendrophthoe falcata* leaves at doses of 100, 200 and 300 mg/Kg as an aqueous suspension in 2% v/v Tween 80. After 1 h, 0.1 mL of 1% w/v carrageenan suspension was injected subcutaneously into plantar surface of the right hind paw. The paw volume was measured using the plethysmometer at 60, 120, 180 and 240 min after carrageenan injection. 

*Effects in cotton pellet granuloma*

The rats were divided into eight groups and each group consisted of six animals. After shaving the fur, the animals were anaesthetized using ketamine. Sterile pre-weighed cotton pellets (20 ± 1 mg) were implanted in the auxiliary region of each rat through a single needle incision. Control (2% v/v aqueous Tween 80 solution, 5 mL/Kg), standard (phenylbutazone, 150 mg/Kg, p.o.), aqueous extracts of *Dendrophthoe falcata *leaves (100, 200, 300 mg/Kg) and DFM (100, 200, 300 mg/Kg) were administered to the respective group of animals for seven consecutive days from the day of cotton pellet implantation. On the eighth day, animals were anaesthetized again; the cotton pellets were removed surgically and made free from extraneous tissues. The pellets were incubated at 37°C for 24 h and dried in an oven at 60°C to constant weight. The increment in the dry weight of the pellets was regarded as a measure of granuloma formation (9). 

Determination of DPPH (1, 1-diphenyl -2-picryl-hydrazyl) radical scavenging activity 

1 mL different concentration of extract solution and standard were taken in different vials. 5 mL of methanolic solution of DPPH (1, 1-diphenyl -2-picryl-hydrazyl) was added, shaken well and the mixture was incubated at 37°C for 20 min. The absorbance was measured against methanol as blank at 517 nm. The absorbance of the DPPH (1, 1-diphenyl -2-picryl-hydrazyl) was taken as the control (10). The antiradical activity percentage can be calculated, using following formula: 

$\mathrm{Antiradical activity}=\frac{\mathrm{Control Abs}-\mathrm{Sample A}}{\mathrm{Control Abs.}}\times 100$

*Anti-lipid peroxidation activity*

*Anti-lipid peroxidation in liver homogenate*

Preparation of liver homogenate: The liver was perfused with ice cold 0.15 M KCl via portal vein. The perfused liver was isolated and 10% (w/v) homogenate was prepared using a tissue homogenizer under ice cold (0-4°C) condition. The homogenate was used to study *in-vitro* lipid peroxidation (11).

A mixture of 0.5 mL of homogenate, 1 mL of 0.15 mL KCl and 0.5 mL of different concentration of drug extracts, was prepared. Lipid peroxidation was initiated by adding 100 µL of 1 mm ferric chloride. The reaction mixture was incubated for 30 min. at 37°C. After incubation, the reaction was stopped by adding 2 mL of ice cold 0.25 N, HCl containing 15% TCA, 0.38% TBA and 0.2 mL of 0.05% butylated hydroxyl toluene (BHT). These reaction mixtures were heated for 60 min at 80^º^C. They were also cooled and centrifuged at 5000 rpm for 15 min. The absorbance of the supernatant was measured at 532 nm against a blank which contained all reagents except liver homogenate and drug. Identical experiments were performed to determine the normal (without drug and ferric chloride) and induced (without drug) lipid peroxidation.

$\mathrm{\%ALP}=\frac{\mathrm{Ferric chloride O.D.}-\mathrm{Sample O.D.}}{\mathrm{Ferric Chloride O.D.}-\mathrm{Normal O.D.}}\times 100$

*Nitric oxide scavenging activity*

Different concentrations of sample solution were prepared in 100 mL volumetric flask. 0.1489 g of sodium nitroprusside (final concentration 5 mm) was added to this and kept for incubation. At different time points, 5.6 mL was taken, 0.2 mL of reagent A was added and kept for incubation at 30°C for 10 min. After incubation, 0.2 mL of Griess reagent was added and kept for incubation at 30°C for 20 min and the absorbance was measured at 542 nm against blank (12).

$\mathrm{\% NO Scavenging activity}=\frac{\mathrm{Control Abs}-\mathrm{Sample A}}{\mathrm{control Abs}}\times 100$

*Estimation of total phenolics and total flavonoids*

The total phenolic of extracts was determined using folin-ciocalteu reagent (13). The extracts (100 µL, three replicates) were mixed with the folin-ciocalteu phenol reagent (0.2 mL), water (2 mL) and sodium carbonate (15% w/v, 1 mL) and the absorbance at 760 nm was measured 2 h after incubation at 50°C for 10 min. The total phenolic was expressed as mg/mL of gallic acid. 

The total flavonoids of extracts were determined through the reported method (14). In brief, the extract was diluted with 80% aqueous ethanol (0.9 mL). Aliquot of 0.5 mL of extract was added to test tube containing 0.1 mL of 10% aluminum nitrate, 0.1 mL of 1 M aqueous potassium acetate and 4.3 mL of 80% ethanol. After 40 min at room temperature, the absorbance was determined at 415 nm with UV spectrophotometer. Total flavonoid content was calculated according to a standard curve established with quercetin as reference. Quercetin and folin-ciocalteu reagent were obtained from Sigma-Aldrich, Germany.

*Statistical analysis*

The results are presented as Mean ± SEM one-way analysis of variance (ANOVA) followed by Dunnett’s t-test for multiple comparisons, was used for statistical evaluation. The p-values less than 0.05 were considered as significant.

## Results

The intraplantar injection of carrageenan in the hind paw induced gradual increase in the edema paw volume in the control group. Aqueous extracts of *Dendrophthoe falcata *leaves and methanolic extract of *Dendrophthoe falcata* leaves at doses of 100, 200 and 300 mg/Kg, significantly (p < 0.01) inhibited edema formation in rat paw, 240 min after the carrageenan challenge (Table 1). The standard drug, Diclofenac sodium at dose 5 mg/Kg, marked reduction in paw edema. Aqueous extracts of *Dendrophthoe falcata *leaves and methanolic extract of *Dendrophthoe falcata* leaves at doses of 100, 200 and 300 mg/Kg significantly (p < 0.01) inhibited granuloma formation (Table 2).

**Table 1:** The effect of *Dendrophthoe falcata L*. Leaves extracts on carageenan –induced rat paw edema.

**Groups (n = 6)**	**Carageenan induced rat paw edema volume in mL (Mean ± SEM) (% inhibition)**				
	**0 (min)**	**60 (min)**	**120 (min)**	**180 (min)**	**240 (min)**
Control, 0.1(mL), 1% Carageenan	1.16 ± 0.005	1.64 ± 0.017	1.94 ± 0.009	2.30 ± 0.011	2.52 ± 0.01
Diclofenac sodium, 5 (mg/Kg)	1.14 ± 0.006	1.18 ± 0.005* ^(28.04)^	1.25 ± 0.009** ^(35.56 )^	1.34 ± 0.010** ^(41.73 )^	1.44 ± 0.019** ^(42.85 )^
DFA, 100 (mg/Kg)	1.15 ± 0.008	1.53 ± 0.010 ^(6.70 )^	1.70 ± 0.008** ^( 12.37)^	1.81 ± 0.010** ^( 21.30)^	2.05 ± 0.15** ^(18..56 )^
DFA, 200 (mg/Kg)	1.19 ± 0.005	1.46 ± 0.015* ^(10..97 )^	1.54 ± 0.012** ^( 20.61)^	1.64 ± 0.016** ^(28.69 )^	1.85 ± 0.13** ^( 26.58)^
DFA, 300 (mg/Kg)	1.13 ± 0.005	1.30 ± 0.007* ^(20.73 )^	1.42 ± 0.011** ^(26.80 )^	1.54 ± 0.012** ^(33.04 )^	1.74 ± 0.009** ^( 30.95)^
DFM, 100 (mg/Kg)	1.20 ± 0.012	1.55 ± 0.011** ^(5.48 )^	1.80 ± 0.015** ^( 7.21)^	2.04 ± 0.009** ^(11.30 )^	2.30 ± 0.008** ^(8.73 )^
DFM, 200 (mg/Kg)	1.18 ± 0.005	1.51 ± 0.16 ^(7.92 )^	1.77 ± 0.007** ^(8.76 )^	1.96 ± 0.008** ^( 14.78)^	2.22 ± 0.008** ^(11..90 )^
DFM, 300 (mg/Kg)	1.18 ± 0.005	1.48 ± 0.015* ^(9.75 )^	1.65 ± 0.012** ^(14.94 )^	1.85 ± 0.012** ^( 19.56)^	1.93 ± 0.007** ^( 23.41)^

DFA: Aqueous extract of *Dendrophthoe Falcata *leaves

DFM: Methanolic extract of *Dendrophthoe Ffalcate *leaves

^* *^: p< 0.01^ *^: p< 0.05 Values are expressed as Mean ± SEM, one-way analysis of variance followed by Dunnette’s multiple comparison -tests.

**Table 2:** The effect of *Dendrophthoefalcata* L. Leaves extract on cotton pellet-induced granuloma in rats

**Groups**	**Dose (mg/Kg)**	**Weight of dry cotton pellet granuloma (mg) (Mean ± SEM)**	**Percentage of inhibition**
Control	Normal Saline 5 (mL/kg) p.o	80 ± 5.8	------
Phenylbutazone	150 (mg/Kg) p.o.	37.2 ± 4.57^**^	53
DFA	100 (mg/Kg) p.o.	50.0 ± 1.15^**^	37
DFA	200 (mg/Kg) p.o.	43.0 ± 2.77^**^	46
DFA	300 (mg/Kg) p.o.	39.0 ± 0.96^**^	51
DFM	100 (mg/Kg) p.o.	56.0 ± 1.18^**^	30
DFM	200 (mg/Kg) p.o.	45.7 ± 3.29^**^	43
DFM	300 (mg/Kg) p.o.	41.0 ± 1.03^**^	48
one-way ANOVA F(df) = 21(5,30) p < 0.0001			

DFA: Aqueous extract of *Dendrophthoe Falcata *leaves

DFM: Methanolic extract of *Dendrophthoe Falcate *leaves

^* *^: p< 0.01

^ *^: p< 0.05

Values are expressed as Mean ± SEM, one-way analysis of variance followed by Dunnette’s multiple comparison -tests.

Phenylbutazone (150 mg/Kg p.o.) – a standard drug-elicited marked reduction in granuloma formation.

In addition, methanolic extract and aqueous extract of *Dendrophthoe falcata* leaves showed potent antioxidant activity in different *in-vitro* models like DPPH (1, 1-diphenyl -2-picryl-hydrazyl) radical scavenging, anti-lipid peroxidation and nitric oxide scavenging activity, having IC_50_ values 77.8, 79.36 and 86.2, 144, 87, 104 mcg respectively. However, methanolic extract of*Dendrophthoe falcata *leaves shows good anti-oxidant activity as compared to aqueous extract of *Dendrophthoe falcata *leaves. In addition, the total phenolic and flavonoid content of aqueous extract of *Dendrophthoe falcata* leaves and methanolic extract of *Dendrophthoe falcata* leaves was found to be 2.12% w/w, 4.39% w/w, 0.31 mg/g and 0.85 mg/g respectively, which plays the major role in controlling antioxidants (15).

## Discussion

The aqueous extracts and methanolic extract of *Dendrophthoe falcata* leaves, significantly suppressed the carrageenan induced rat paw edema 4 h after carrageenan challenge. Carrageenan induced rat paw edema is commonly used as an experimental animal model for evaluation of anti-inflammatory potential of natural products(8)and is believed to be biphasic. The initial phase is due to the release of histamine, serotonin and kinin in the first hour after administration of carrageenan. The more pronounced second phase is attributed to release of bradykinin and prostaglandin. The cotton pellet granuloma bioassay is considered as a model for studies of chronic inflammation and is considered as a typical feature of established chronic inflammatory reaction(16). The aqueous extracts of *Dendrophthoe falcata* leaves and methanolic extract of *Dendrophthoe falcata* leaves exhibited significant reduction of granuloma formation in rats in the cotton pellet-induced granuloma. This means that aqueous extracts and methanolic extract of *Dendrophthoe falcata* leaves may be effective in chronic inflammatory conditions. The result of the present study indicates that the crude fraction of aqueous and the methanol extracts of *Dendrophthoe falcate* possess significant anti-inflammatory activity. 

Oxidative stress is major upstream in component signaling-cascade involved in inflammatory response, stimulating adhesion molecules and chemoactractant production. Thus it will be relevant to investigate theantioxidant potential of hemiparasite, it is evident that methanolic extract of *Dendrophthoe falcata* leaves was potent antioxidant over aqueous extracts of *Dendrophthoe falcata *leaves with IC_50 _of 77.8, 79.36 and 86.2 µg/mL for DPPH (1, 1-diphenyl -2-picryl-hydrazyl) radical scavenging activity, antilipid peroxidation and nitric oxide scavenging activity, respectively. Thus antioxidant data, demonstrates that methanolic extract of *Dendrophthoe falcata*leaves is a more potent antioxidant than aqueous extracts of *Dendrophthoe falcata *leaves and this observation was further supported by total phenolic and total flavonoid content of methanolic extract of *Dendrophthoe falcata*leaves and aqueous extracts of *Dendrophthoe falcata *leaves. However, we observe that the higher antioxidant potential of hemiparasite is attributed to its own flavonoid content and the transferred phenolic, hardly gives significant rise in antioxidant activity. The high concentration of phenolics, appeared to be a general feature of parasitic angiosperms (17) and it was evident that the hemiparasite had a higher flavonoids content than host plant, *Mangifera indica*. Considering all these observations we come the fact that anti-inflammatory activity of methanolic extract of *Dendrophthoe falcata* leaves in both acute and chronic inflammatory conditions, is mostly attributed to its flavonoids and subsequently its anti-oxidant potential since almost every group of flavonoids has antioxidant property and flavonoids are known to participate in the cellular antioxidant network (18). 

Thus from the results presented here, aqueous extract of *Dendrophthoe falcata *leaves was found to be potent over methanolic extract of *Dendrophthoe falcat*a leaves in acute and chronic inflammation but aqueous extracts of *Dendrophthoe falcata* leaves was less potent as an antioxidant agent over methanolic extract of *Dendrophthoe falcata* leaves. 

Taken together, the present study confirms the anti-inflammatory and the anti-oxidant potential of plant which not only rationalizes the some of ethanomedicinal claims, but also identified a potential candidate for further investigation, especially for chronic inflammatory conditions such as rheumatoid arthritis. A further research is in progress to identify the biomolecules responsible for the anti-inflammatory and antioxidant activities.
